# Implementation, relevance, and virtual adaptation of neuro-oncological tumor boards during the COVID-19 pandemic: a nationwide provider survey

**DOI:** 10.1007/s11060-021-03784-w

**Published:** 2021-06-11

**Authors:** Niklas Schäfer, Elisabeth Bumes, Fabian Eberle, Viola Fox, Florian Gessler, Frank A. Giordano, Juergen Konczalla, Julia Onken, Malte Ottenhausen, Moritz Scherer, Matthias Schneider, Hartmut Vatter, Ulrich Herrlinger, Patrick Schuss

**Affiliations:** 1 Division of Clinical Neuro-Oncology, Department of Neurology, University Hospital Bonn, Venusberg-Campus 1, 53127 Bonn, Germany; 2 Neuro-Oncology Center, Center of Integrated Oncology (CIO) Bonn, University Hospital Bonn, Bonn, Germany; 3 Department of Neurology and Wilhelm Sander-NeuroOncology Unit, Regensburg University Hospital, Regensburg, Germany; 4 Department of Radiation Oncology, University Hospital Marburg, Marburg, Germany; 5 Lung Cancer Center Köln-Merheim, Cologne, Germany; 6 Department of Neurosurgery, University Medicine Rostock, Rostock, Germany; 7 Department of Radiotherapy and Radiation Oncology, University Hospital Bonn, Bonn, Germany; 8 Department of Neurosurgery, University Hospital Frankfurt, Frankfurt, Germany; 9 Department of Neurosurgery, Charité-Universitätsmedizin Berlin, corporate member of Freie Universität Berlin, Humboldt-Universität Zu Berlin, Berlin, Germany; 10 Berlin Institute of Health, Berlin, Germany; 11 German Cancer Consortium (DKTK), Partner Site Berlin, CCCC (Campus Mitte), Berlin, Germany; 12 Department of Neurosurgery, University Hospital Mainz, Mainz, Germany; 13 Department of Neurosurgery, University Hospital Heidelberg, Heidelberg, Germany; 14 Department of Neurosurgery, University Hospital Bonn, Bonn, Germany

**Keywords:** Neuro-oncology, Tumor board, Virtualization, COVID-19

## Abstract

**Purpose:**

Neuro-oncology tumor boards (NTBs) hold an established function in cancer care as multidisciplinary tumor boards. However, NTBs predominantly exist at academic and/or specialized centers. In addition to increasing centralization throughout the healthcare system, changes due to the COVID-19 pandemic have arguably resulted in advantages by conducting clinical meetings virtually. We therefore asked about the experience and acceptance of (virtualized) NTBs and their potential benefits.

**Methods:**

A survey questionnaire was developed and distributed via a web-based platform. Specialized neuro-oncological centers in Germany were identified based on the number of brain tumor cases treated in the respective institution per year. Only one representative per center was invited to participate in the survey. Questions targeted the structure/organization of NTBs as well as changes due to the COVID-19 pandemic.

**Results:**

A total of 65/97 institutions participated in the survey (response rate 67%). In the context of the COVID-19 pandemic, regular conventions of NTBs were maintained by the respective centers and multi-specialty participation remained high. NTBs were considered valuable by respondents in achieving the most optimal therapy for the affected patient and in maintaining/encouraging interdisciplinary debate/exchange. The settings of NTBs have been adapted during the pandemic with the increased use of virtual technology. Virtual NTBs were found to be beneficial, yet administrative support is lacking in some places.

**Conclusions:**

Virtual implementation of NTBs was feasible and accepted in the centers surveyed. Therefore, successful implementation offers new avenues and may be pursued for networking between centers, thereby increasing coverage of neuro-oncology care.

**Supplementary Information:**

The online version contains supplementary material available at 10.1007/s11060-021-03784-w.

## Introduction

Treatment of patients with cancers affecting the central and peripheral nervous system is complex and requires a coordinated team of specialists. Multidisciplinary tumor boards (MTBs) form the foundation for highly specialized (neuro)-oncology care and the continuous maintenance of the highest quality in cancer care [1]. The benefits of MTBs include efficient collaboration of multiple providers, communication between treatment teams, continuous education, increased adherence to treatment guidelines, and access to clinical trials [2, 3]. However, MTBs not only provide an opportunity for consensus building on the best possible treatment regimens for individual patients, but also make a significant contribution to collegial adherence in individual case decisions. However, a study by Snyder et al. showed a high degree of heterogeneity in the implementation, proceeding, and documentation of such MTBs [4]. Going beyond the implementation of general MTB, Robin et al. recommend the establishment of a multidisciplinary brain tumor board led by neuro-oncologists for patient-centered neuro-oncology treatment planning and management to address the multiple demands in neuro-oncology patients. [5]. Appropriately, Snyder and colleagues state here that the implementation and delivery of neuro-oncology MTBs, including those at nonacademic centers, is critical for nationwide quality assurance of neuro-oncology patient care [4]. In addition, highly specialized neuro-oncology tumor boards (NTB) focusing solely on neuro-oncological patients cannot easily be established at every center. Nevertheless, the survival advantage of neuro-oncological patients treated at high-volume and/or academic centers seems evident [6, 7]. The survey by Snyder et al. revealed that although the academic tumor centers polled had a desire to review external cases, only a quarter also experienced involvement from affiliated satellite centers [4]. To address the issue of nonparticipation of external centers, both teleconferencing options and increased use of virtual platforms have been proposed but rarely implemented to date [8, 9]. However, in the context of the COVID-19 pandemic, numerous efforts to increase digitalization/virtualization, particularly in healthcare, have accelerated [10, 11]. This digitalization leap has not just created new challenges but, conversely, is also fostering new opportunities for expert networking in (neuro-) oncological tumor care [12].

The aim of the study was to identify the implementation modalities in academic and non-academic hospitals regarding NTBs in Germany in order to use this knowledge to further improve current practice and, if possible, to take advantage of the trends towards increasing digitalization.

## Methods

### Survey population—neuro-oncological specialty centers

The surveyed neuro-oncology centers in Germany were identified via Germany’s leading provider transparency portal WeisseListe.de (WL.de). The portal WL.de has become the largest public portal for quality reports in healthcare in Germany [13]. The collected information originates from the statutory quality reports of over 2000 hospitals in Germany [14]. Since these quality reports are also based on the hospitals' accounting data, it was possible to record the number of reported brain tumor cases for each hospital using ICD-10 coding. For each hospital reporting more than 50 cases with ICD-10 code C71 (malignant disease of the brain) in a year, the authors' team manually identified one person responsible for neuro-oncology therapy using address lists. In this way, only one report per hospital took place with regard to the survey.

### Survey questionnaire

Adhering to the design of a cross-sectional study, an online survey is created via a web-based platform (SurveyMonkey Inc.; San Mateo, California, USA; www.surveymonkey.com). The survey consisted of 24 questions formulated after discussion among the authors (supplementary appendix). The questions were grouped into 4 categories: (1) structure, (2) function/implementation, (3) changes due to the COVID-19 pandemic, and (4) impact of NTB on clinical practice. The questions on structure included information on the surveyed institutions and the format of NTB. Questions on the function/implementation of the NTB addressed information on meeting tasks, activities, and staff composition as well as the respective implementation of the NTB in the participating centers. Changes caused by the COVID-19 pandemic were asked in a separate section (with an explicit focus on the expected increase in virtualization). Impact questions addressed the individual value of meetings as well as barriers. The survey was conclusively reviewed internally by a multidisciplinary group of physicians involved in neuro-oncology care at the University Hospital Bonn. The study was approved by the Institutional Review Board of the Medical Faculty of Bonn (no. 063/21).

### Data collection

The survey was sent by email in March 2021 to the responsible medical staff involved in neuro-oncology at each hospital that had previously been identified using the described pathway. A total of 97 hospitals in Germany were contacted. No rewards or incentives for participation in the survey were offered, and those who refused to participate and/or did not complete the survey (more than three questions were missing) were considered non-responders. After one week, a reminder was sent in the same way to increase the response rate. Unique visitors were identified based on IP addresses and were used to prevent multiple entries from the same individual. The survey was available for a total duration of 3 weeks.

### Data analysis

Survey responses were collected, downloaded, and converted into a dataset for further analysis. Summary statistics, simple and stratified, were compiled using SPSS (version 25, IBM Corp., Armonk, NY).

## Results

### Respondent characteristics

**Fig. 1:**
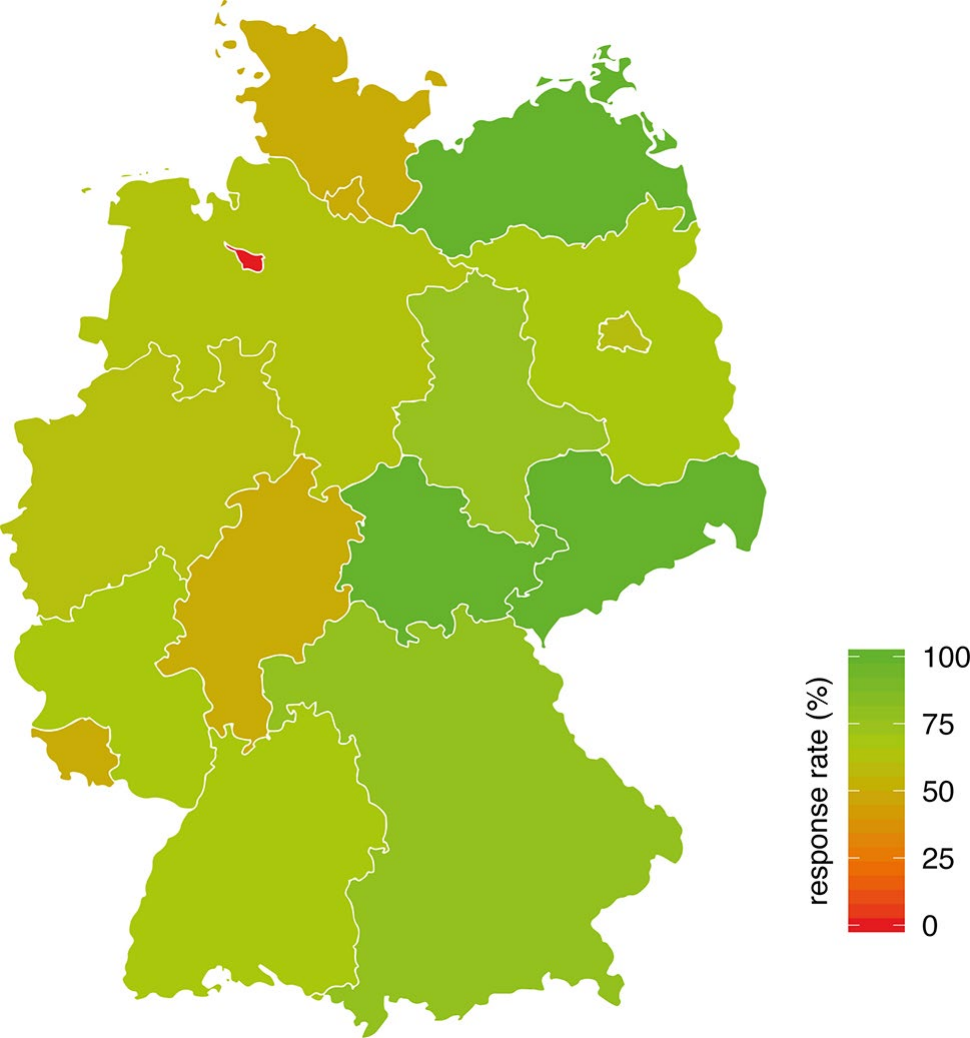
Proportion of responding centers per federal state in Germany

Of the 97 centers invited, 65 (response rate 67%) responded to this survey. With the exception of one federal state, ≥ 50% of centers per federal state in Germany were represented (Fig. 1). Of the responding centers, 53% were a part of a university hospital, 29% were a part of a municipal hospital, and 18% were a part of a hospital with a private carrier. Overall, 5% of surveyed centers stated that they did not implement or were not affiliated with an NTB.

### Characteristics of the neuro-oncological tumor boards

All centers that implemented NTB did so on a weekly schedule. The practice of conducting an NTB had been established for > 3 years in 89% of the participating centers. 10% of centers had been conducting or participating in an NTB for 1–3 years, whereas 2% had been doing so for < 1 year. Details on the participating specialties of NTBs are given in Table 1.

**Table 1:** Participating specialties in neuro-oncological tumor boards

Participating disciplines	(%)
Neurology/neuro-oncology	77
Neurosurgery	98
Radiation oncology	89
Medical oncology	97
(Neuro-) radiology	98
(Neuro-) pathology	89
Nuclear medicine	25
Dermatology	15
ENT/oral and maxillofacial surgery	20
Palliative medicine/care	1
Oncology nursing	5
Social worker	2
Study nurse/clinical trial personal	14

Regarding the diagnoses discussed in the NTB, all centers reported discussing patients with primary brain tumors (100%). Brain metastases were also discussed in 95% of the centers and spinal tumor cases in 86%. 47% of the participating centers also consulted on the treatment of paraneoplastic disease within their NTB. 86% reported to perform collaborative preliminary discussion of cases with lesions of unknown etiology. Presentation of the cases to be discussed is performed in 79% by the treating physician, in 56% also by a resident physician, and in only 5% by recitation of text-only information about the patient. 84% of responding centers reported receiving detailed information about patient comorbidities as part of the case presentation. In 82% of the cases, the NTBs also directly address the inclusion possibilities of potential clinical trials.

Case presentation includes active demonstration of radiological imaging in 98%, and description of histological findings by (neuro)pathology in 82%. In 89% of NTBs, the results of additional molecular pathology investigations are also part of the individual case discussion. 21% state that, especially in rare cases, a summary of the current research and/or a literature review is included in the case briefing. In 20%, the demonstration of clinical cases is additionally complemented by histopathological images.

Documentation of NTB consultation results is done digitally in the patient’s record in 91% of centers, including a separate report in 27% of cases, while in 9% of cases documentation is handled in the paper-based medical record. 73% of responding centers conduct regular morbidity and mortality (M&M) conferences focusing on patient safety and quality improvement within their NTB, of which 88% do so at least twice a year.

### Changes during the COVID-19 pandemic

The circumstances of the COVID-19 pandemic, including the contact restrictions required, have led to increased use of virtual technology in 68% of participating centers. 23% of centers are now operating their NTB completely virtually, while 48% are using a partially virtual environment but are responding to the pandemic's sanitation requirements with either larger locations, fewer people present, or increased sitting distance. The distribution of the utilized software for virtual implementation of NTB is as follows: 17% Zoom or Cisco Join/Webex, respectively, 13% Skype for business, and 8% Microsoft Teams. The remaining used other or institutional solutions.

In free-text response options regarding subjective benefits of virtual NTB, the centers that have completed a virtual NTB conversion advocate improved integrability into daily clinical practice, significant time savings, better integration of external specialties/colleagues, higher numbers of participants, and significantly enhanced flexibility.

### Value of neuro-oncological tumor board

All respondents consider the value of NTB to be the significant benefit of improving communication among medical colleagues (100%). 88% consider the NTB as an opportunity to jointly achieve optimal/improved standardization and quality of care. 78% of the responding centers perceive an advantage in the continuous medical education stimulated by the NTB. 62% of the participants thought that the interdisciplinary meeting of NTB enables more treatment options for the individual patient. 50% also regard the possibility of increased recruitment of patients for clinical neuro-oncological studies as an advantage of NTB.

The major obstacle to conducting a weekly NTB is perceived by 35% of respondents to be the high number of cases, while 25% consider it difficult to integrate the NTB into their daily clinical duties, 28% complain about a lack of support from the clinical administration and/or information technology (IT) services, whereas 27% identify the absence of individual specialty departments during the NTB and/or within their center as a major constraint. According to respondents, 10% of centers experience scheduling conflicts between multiple MTBs. Within the scope of the free-text answers, complaints are found about the long duration of the weekly NTB, the resulting inconsistency with the stipulated work hours, and the sometimes deficient preparation/presentation of the cases to be discussed.

## Discussion

Given the increased sub-specialization in the context of increasingly individualized tumor management, the establishment of specialized tumor boards (such as neuro-oncological tumor boards) seems worthwhile [15, 16]. The respective benefits for both treatment and outcome of the patient, as well as the continuing education and training for the treating physicians along with the continuous self-reflection on applied treatment strategies in addition to the possibility of enrolling patients in clinical trials are proven advantages of NTBs [4, 17, 18]. Nevertheless, a continuous centralization of treatment can also be observed for neuro-oncological conditions, with a consequential reorganization of the hospital landscape towards the development of regional specialty centers/large-volume centers [19]. In addition to lower morbidity and mortality after (neuro)surgical procedures, improvements in overall survival have been demonstrated for various cancer types in large-volume centers due to the often improved inner-hospital multidisciplinary networking [7, 20–22]. Furthermore, corresponding accrediting authorities also set certain thresholds for the number of cases to be treated as part of the certification process for specialty cancer centers. To what extent this progressive centralization of neuro-oncology treatment can also ensure nationwide coverage is as yet unknown.

For this reason, our survey was addressed to German institutions with experience in neuro-oncology based on the number of diagnoses of malignant primary brain tumors per year. Given the thorough and stringent selection of survey participants, as well as the response rate of more than 60%, we believe that the results presented here are robust for representative interpretation. Noteworthy, many of the respondents were non-academic institutions, mirroring a broad neuro-oncological care network in Germany given the low incidence rates of malignant primary brain tumors. Among the national facilities surveyed, NTBs are generally accepted and represent a worthwhile component of daily clinical routine in the view of the survey participants. Respondent institutions reported a high level of experience with NTBs, with 89% having established NTBs for more than 3 years. Only 5% of the responding centers have currently no NTBs implemented. This finding is underlined by the way cases are presented during these NTBs. The reported predominant participation and presentation by residents/treating physicians may indicate a true accountability of treating physicians and allows for detailed case discussions as well as study recruitment. Further, the estimated value of NTBs may increase willingness to participate. The vast majority rated the opportunities for communication, implementation of optimal patient care, and continuing medical education as beneficial. As expected, participation in NTBs is perceived to be time-consuming and may therefore interact with other duties in clinical practice.

### COVID-19 pandemic leading towards virtualization of NTBs

The tremendous impact on daily living during the COVID-19 pandemic resulted primarily in shifts in medical care [23–26]. However, the results of our survey impressingly demonstrate that NTBs were maintained during this period. This indicates the great efforts of local institutions and departments at this time, as well as the medical need for NTBs. Moreover, this occurred without a lower participation rate of individual disciplines, as could be demonstrated by the outstanding reported participation rate of neurosurgeons, radiation oncologists, medical oncologists and (neuro-)radiologists throughout this straining pandemic. A reason for this achievement may be the successful transition from face-to-face meetings to virtual meetings. This switch was reported as technically feasible and even resulted in a better integration of NTBs in daily practice routine. Interestingly, given the plethora of applicable software for virtual NTBs, the majority of institutions chose to use software from Zoom, Cisco, and Skype. In doing so, the virtual nature of the meetings seemed to facilitate the participation of the various disciplines, which would mean a significant benefit and increase in quality of NTBs held virtually. Another advantage of a virtual implementation of NTBs seems to be easier NTB participation of local and/or external guests, which allows for more in-depth individual case discussion and also cross-regional networking of all participating physicians [27]. Nevertheless, increasing virtualization also poses a challenge to strict compliance with data protection laws. Particularly in the case of cross-regional NTBs, this requires close coordination between the respective data protection officers in accordance with the prevailing federal state data protection laws.

### Value and relevance of NTBs

The multidisciplinary approach in neuro-oncology implies non-delegable tasks of neurosurgeons and radiation oncologists as well as shared topics such as diagnostics and/or medical treatment, supportive and palliative care. Between these different disciplines, a leadership role for patients and caregivers is needed to ensure fixed contact partners and a managing/coordinating department. Especially patients with primary brain tumors as well as their caregivers might need a designated site for contact due to the high amount of psycho-social distress and impairment in physical and cognitive functioning [28]. Neuro-oncological neurologists, as representatives of a non-interventional discipline, may be predisposed to this role. As a noble goal, they might hold the reins, thereby coordinating necessary further medical/surgical consultations and providing additional (neurologically skilled) supportive therapy to assist patients and their families throughout the course of their neuro-oncological disease. Obviously, these efforts and demand of great commitment could be supplanted by either one of the partnering disciplines. Nevertheless, only 4 of 5 participating institutions report that neurologists attend their NTBs, which makes increased enforcement within neuro-oncology by neurology specialists desirable given the medical care that must be provided to patients with tumors of the central nervous system.

Apart from patient management, the majority of respondents agree that the NTBs established at their centers contribute to improved physician communication, better interdisciplinary networking, and thus more optimal treatment of the patients entrusted to them. Resulting from the necessary contact restrictions in the context of the COVID-19 pandemic, the increased virtualization of NTBs promises to have a positive effect on improving attendance even for specialties not residing at the same hospital. Furthermore, due to the growing centralization in the healthcare system, a significantly improved integration of low-volume/remote hospitals might become feasible and thus contribute to a comprehensive, highly-specialized and optimal treatment of patients with CNS tumors.

### Limitations

The main limitation of the present work that goes common to all surveys is the inability to generalize the results based on the selected surveyed sample. Due to the described selection procedure of the recipients of the survey, there is also a risk of selection bias. Nevertheless, the selection approach reduces the risk of multiple responses per center, and the overall survey coverage achieved is likely to reflect a reliable sentiment regarding the implementation of NTBs in Germany. Furthermore, the selection of one contact person per center naturally negates the discipline-specific characteristics, which, on the other hand, were not addressed by the survey itself.

## Conclusions

Increasing centralization in the healthcare system also affects patients suffering from neuro-oncological tumors. The enormous efforts of healthcare providers in the context of the COVID-19 pandemic, including the augmented virtualization of neuro-oncological tumor boards, could help to implement optimal care for neuro-oncological patients even in remote hospitals and thus nationwide.

## Supplementary Information

Below is the link to the electronic supplementary material.

Supplementary file1 (DOCX 17 kb)

## Data Availability

The datasets generated during and/or analysed during the current study are available from the corresponding author on reasonable request.

## Code availability

A web-based platform was used provided by SurveyMonkey Inc. (San Mateo, California, USA; www.surveymonkey.com). For data analysis we used SPSS version 25 (IBM Corp., Armonk, NY).
