# Supplementary material for: Implementation, relevance, and virtual adaptation of neuro-oncological tumor boards during the COVID-19 pandemic: a nationwide provider survey
Source: J Neurooncol. 2021 Jun 11;153(3):479–85. doi: 10.1007/s11060-021-03784-w (PMC8192684; doi:10.1007/s11060-021-03784-w)
Supplement: Supplementary file 1 — Supplementary file1 (DOCX 17 kb) [file 11060_2021_3784_MOESM1_ESM.docx]

**Questions for neuro-oncological tumor board (NTB) survey**

(translated from German)

1. Which state does your institution belong to?
2. Which category does your institution belong to?
   - University hospital
   - State hospital
   - Private sponsor
3. How often does your institution hold a NTB?
   - weekly
   - twice a month
   - monthly
   - on demand
   - n/a *(automatic forwarding to question XY)*
4. How long has regular NTB been taking place at your institution at the current level?
   - < 1 year
   - 1-3 years
   - >3 years
5. Who regularly participates in the NTB?
   - Neurology/neuro-oncology; neurosurgery; (neuro-)radiology; radiotherapy; oncology; (neuro-)pathology; ENT; oral and maxillofacial surgery; dermato-oncology; nuclear medicine; palliative care; oncology nursing; social service staff; MTA/study nurse; free response option.
6. In which of the following ways does NTB implementation take place at your institution?
   - Face-to-face meetings on premises
   - Partly virtual
   - Entirely virtual
   - Teleconference
7. What software do you use for the virtual implementation of the NTB?
8. Has there been an increased use of virtual technology during the Covid 19 pandemic?
9. To what extent has your NTB infrastructure changed in the context of the Covid 19 pandemic?
   - now fully virtual
   - now partly virtual
   - less frequent meetings
   - larger premises
   - free-text response option
10. In your opinion, what are the advantages of a planned/successful virtual conversion of the NTB?
11. What diagnoses are discussed as part of your NTB?
    - Primary brain tumors
    - brain metastases
    - spinal processes
    - paraneoplastic processes
    - neoplasms of unknown behavior (pre-therapeutic discussion)
12. Who usually presents the patients to be discussed?
    - Treatment leader/specialist
    - assistant physician
    - MTA/documentation assistant
    - information of the request text
13. Do you generally receive more detailed information about comorbidities as part of the presentation?
14. Does the NTB meeting review the possibility of inclusion in clinical trials?
15. By which of the following is the case discussion supported in your NTB?
    - active demonstration of radiologic images
    - description of radiologic findings without active demonstration of images
    - active demonstration of histologic sections
    - description of histologic findings without active demonstration of sections
    - molecular pathology findings
    - genetic studies
    - photographs/videos of clinical findings
    - literature review
    - opportunity for free text response
16. Is there a possibility for external colleagues to present their own patients in your NTB?
    - yes, by mail
    - yes, virtual participation/telephone
    - no
17. Is further radiation therapy treatment usually provided at your institution?
18. Is further chemotherapy treatment usually administered at your institution?
19. Which of the following aspects of post-treatment care are considered as part of case discussions in your NTB?
    - preparing for discharge to home
    - nutrition
    - managing epilepsy
    - psycho-oncology outpatient services
    - wound care
    - free text response option
20. How is the documentation of the consulting results done?
    - digital patient file
    - paper file
    - separate report of NTB results
    - free text response option
21. Do you hold regular morbidity & mortality (M&M) conferences within your NTB meetings?
    - no
    - < 2 / year
    - twice a year
    - > 2 / year
22. Do you receive CME points for participating in your NTB?
23. What do you see as the key benefits of regular NTB meetings?
24. In your opinion, what aspects make it challenging to implement an NTB at your institution?
